# First Isolation and Genome Characterization of *Bocaparvovirus carnivoran 1* (CBoV-1) from a Dog with Acute Hemorrhagic Diarrhea in Mexico

**DOI:** 10.3390/v18101064

**Published:** 2026-09-25

**Authors:** Armando Busqueta-Medina, Ramiro Ávalos-Ramírez, Diana Elisa Zamora-Ávila, Víctor Eustorgio Aguirre-Arzola, Juan Francisco Contreras-Cordero, Domenica Cedillo-Rosales, Sibilina Cedillo-Rosales

**Affiliations:** 1 Universidad Autónoma de Nuevo León, Facultad de Medicina Veterinaria y Zootecnia, Cuerpo Académico de Epidemiología Veterinaria, Departamento de Virología, Campus Ciencias Agropecuarias, Ave. Francisco Villa s/n, Ex-Hacienda el Canadá, General Mariano Escobedo C.P. 66054, Nuevo León, Mexico; armando.busquetamdn@uanl.edu.mx (A.B.-M.); diana.zamoravl@uanl.edu.mx (D.E.Z.-Á.); 2 Universidad Autónoma de Nuevo León, Facultad de Agronomía, Campus Ciencias Agropecuarias, Ave. Francisco Villa s/n, Ex-Hacienda el Canadá, General Mariano Escobedo C.P. 66054, Nuevo León, Mexico; victor.aguirrearz@uanl.edu.mx; 3 Universidad Autónoma de Nuevo León, Facultad de Ciencias Biológicas, Laboratorio de Inmunología y Virología, Ave. Universidad, S/N, Ciudad Universitaria, San Nicolás de los Garza C.P. 66450, Nuevo León, Mexico; juan.contrerascr@uanl.edu.mx; 4 Universidad de Nueva Extremadura (UVNE), Coordinación de la Facultad de Medicina Veterinaria, Av. Manuel Ordóñez #140, Col. Centro, Santa Catarina C.P. 66350, Nuevo León, Mexico; mvz.domenicacedillor@gmail.com

**Keywords:** canine bocavirus, *Bocaparvovirus carnivoran 1*, CBoV-1, phylogenetic analysis, hemorrhagic diarrhea, Mexico

## Abstract

The occurrence of acute hemorrhagic diarrhea in dogs has prompted investigation of emerging enteric viruses, including *Bocaparvovirus carnivoran 1* (CBoV-1), which has been associated with respiratory, gastrointestinal, and reproductive disorders in dogs. In this study, CBoV-1 was successfully isolated in MDCK cells from a clinical sample obtained from a dog with acute hemorrhagic diarrhea. A CBoV-1 genome sequence (4973 bp) was obtained from isolate MX-ABM30 and deposited in GenBank under accession number PQ683622. Phylogenetic analysis revealed a close genetic relationship between MX-ABM30 and CBoV-1 sequences from the United States, with 98% nucleotide identity. A comparative analysis identified six missense mutations across the genome, resulting in four amino acid substitutions in NS1 (K176R, K182N, P669S, and I680L) and two in VP2 (S200G, S536A). This study represents the first report of isolation and characterization of CBoV-1 in Mexico, providing preliminary data on the genetic diversity of this virus in the region. These findings highlight the need for continued surveillance of CBoV-1 and other emerging enteric viruses in canine populations.

## 1. Introduction

Bocaviruses are classified within the family *Parvoviridae*, subfamily *Parvovirinae*, and genus *Bocaparvovirus*, according to the International Committee on Taxonomy of Viruses (ICTV). Unlike other parvoviruses, bocaviruses are characterized by possessing three open reading frames (ORFs) [1]. ORF1 encodes the nonstructural protein NS1, essential for viral replication [2]. ORF2 encodes the structural proteins VP1, VP2, and VP3, which form the viral capsid [3,4,5]. ORF3, located between ORF1 and ORF2, is unique to bocaviruses. It encodes the nuclear phosphoprotein NP1, which regulates the expression of capsid proteins, viral replication, and, in some bocaviruses, the host immune response [6,7,8,9,10]. To date, three species of bocavirus have been described in domestic dogs, associated with different clinical manifestations. *Bocaparvovirus carnivoran 1* (CBoV-1), formerly known as canine minute virus (CnMV), was the first bocavirus identified in dogs in 1970 [11]. Most CBoV-1 infections are considered subclinical; however, it has been associated with severe respiratory disease in puppies [12] and with reproductive and gastrointestinal disorders in adult dogs [13,14,15,16]. *Bocaparvovirus carnivoran 2* (CBoV-2) was first identified in 2012 and shares less than 64% of amino acid identity with CBoV-1 across all viral proteins [17]. CBoV-2 has been associated with severe gastroenteritis and interstitial pneumonia in young and adult dogs [18,19,20]. Finally, *Bocaparvovirus carnivoran 3* (CBoV-3) was identified in 2013 in a dog’s liver with hemorrhagic gastroenteritis and shared 47% and 57% nucleotide identity with CBoV-1 and CBoV-2, respectively [21]. That remains the only reported case of CBoV-3 worldwide, and the virus has not been formally recognized by ICTV.

Although canine parvovirus type 2 (CPV-2) remains the principal etiological agent associated with hemorrhagic diarrhea in dogs [22,23], the emerging recognition of canine bocavirus in gastrointestinal disease has highlighted the need to study its epidemiology and genetic diversity. In America, CBoV-1 had been reported only in the United States and Canada [24,25,26,27]; however, a recent study in Chile detected the virus in 7.5% of dogs, indicating its circulation in Latin America [28]. Given the limited data on canine bocavirus in Mexico, this study reports the isolation and genome characterization of CBoV-1 from one clinical case, representing the first report of this virus in Mexico. 

## 2. Materials and Methods

### 2.1. Clinical Samples and Initial Processing

A fecal sample from a 3-month-old, unvaccinated male Pitbull was submitted to the Veterinary Virology Laboratory, Faculty of Veterinary Medicine, Universidad Autónoma de Nuevo León for viral detection. The patient presented with acute vomiting and severe hemorrhagic diarrhea. Coproparasitological evaluation ruled out parasitic co-infections. Due to rapid progression of the clinical signs, the patient died shortly after presentation, and no medical treatments were administered prior to sampling. The sample tested positive for canine parvovirus type 2 (CPV-2) and negative for canine coronavirus (CCoV) and Giardia spp. via an immunochromatographic CPV/CCV/Giardia Ag Kit (Bionote, Zapopan, Jalisco, Mexico). One milliliter of fecal sample was homogenized with 4 mL of 0.9% sodium chloride (NaCl) solution (1:5 dilution) and centrifuged at 3000× *g* for 5 min. The supernatant was divided into two aliquots: one for viral isolation and one for DNA extraction and PCR, both stored at −20 °C until use. For viral isolation, the clarified supernatant was diluted 1:10 in phosphate-buffered saline (PBS), centrifuged at 3000× *g* for 10 min, and the resulting supernatant was filtered through a 0.22 µm polyethersulfone (PES) membrane filter (Millipore Sigma, Burlington, MA, USA).

### 2.2. Viral Isolation

MDCK cells (ATCC^®^ CCL-34™) at 60–70% confluence in 24-well plates were washed with 200 µL of DMEM supplemented with L-glutamine and sodium pyruvate (Gibco™, Grand Island, NY, USA) and inoculated with 100 µL, 25 µL, and 5 µL of the filtered sample to minimize cellular toxicity. After 1 h incubation at 37 °C in 5% CO_2_ (MCO-19AIC(UV) incubator, Panasonic Healthcare Co., Ltd., Tokyo, Japan), the inoculum was removed and replaced with 400 µL DMEM supplemented with 5% fetal bovine serum (FBS) (Gibco™, Thermo Fisher Scientific, Waltham, MA, USA) and 1% Antibiotic–Antimycotic (100X; penicillin, streptomycin, and amphotericin B) (Gibco™ Thermo Fisher Scientific). Plates were incubated at 37 °C in a humidified atmosphere with 5% CO_2_ and observed daily for 7 days. After the cytopathic effect (CPE) was observed, two additional blind passages were performed (three passages total). All wells with CPE were tested by endpoint PCR for CPV-2, canine circovirus (CCV), canine bocavirus type 1 (CBoV-1), and type 2 (CBoV-2). The CBoV-1 isolate obtained was designated MX-ABM30 (stands for MX = Mexico; ABM = Armando Busqueta Medina; 30 = isolate number 30).

### 2.3. DNA Extraction and Diagnostic PCR

Total DNA was extracted from the original clinical sample and from culture supernatants using the E.Z.N.A.^®^ Tissue DNA Kit (Omega Bio-Tek, Norcross, GA, USA) according to the manufacturer’s instructions. PCR assays were performed using Platinum™ PCR SuperMix (Invitrogen™, Thermo Fisher Scientific) with primers and conditions previously reported by our team (Table 1) [29,30]. Both the initial clinical sample and the culture supernatants were analyzed for CPV-2, canine circovirus (CCV), CBoV-1, and CBoV-2.

### 2.4. Whole Genome Amplification and Sequencing

To amplify the complete genome of CBoV-1, four overlapping primer pairs were designed with the Primer-BLAST tool (version 2.5.0) [31], using as reference CBoV-1 sequences retrieved from the GenBank database (National Center for Biotechnology Information, Bethesda, MD, USA). DNA extracted from the CBoV-1 isolate MX-ABM30 was used as a template. Primers were synthesized by Integrated DNA Technologies (Coralville, IA, USA). Detailed primer specifications are provided in Table 2.

PCRs were performed under the following conditions: an initial denaturation cycle at 94 °C for 5 min, followed by 40 cycles with three temperature steps, denaturation at 94 °C for 30 s, primer annealing at 60 °C for 30 s, and extension at 72 °C for 1 min, followed by a final extension cycle at 72 °C for 10 min and 4 °C for 1 min. The resulting fragments were purified using the E.Z.N.A.^®^ Gel Extraction Kit (Omega Biotek, Norcross, GA, USA) and were Sanger sequenced by Psomagen (Rockville, MD, USA).

### 2.5. Phylogenetic Analysis

CBoV-1 genome was determined by sequencing the four overlapping PCR amplicons in both forward and reverse directions. Chromatograms were evaluated for quality, and the overlapping reads were aligned and assembled to generate the final consensus sequence using SeqBuilder Pro from the DNASTAR Lasergene v.15 software suite (DNASTAR, Inc., Madison, WI, USA). The resulting sequence was compared with complete genome sequences of CBoV-1, CBoV-2, and CBoV-3 available in the GenBank database (National Center for Biotechnology Information, Bethesda, MD, USA). Sequence data are presented in Table S1. Multiple sequence alignment was carried out with the Clustal W algorithm [32]. Genetic distances were calculated using the Kimura 2-parameter model [33]. Dendrograms were constructed using the Neighbor-Joining method [34], and the robustness of the inferred topologies was evaluated by bootstrap analysis with 1000 replicates [35].

## 3. Results

### 3.1. Viral Isolation

Although CPV-2, CCV, and CBoV-1 were PCR-detected in the clinical sample, only CBoV-1 was successfully isolated in MDCK cells, as viral replication of CPV-2 and CCV could not be confirmed by PCR in the culture supernatants following blind passages. CPE induced by CBoV-1 was characterized by cell lysis, morphological changes including cell rounding, and detachment of the cell monolayer. These changes were observed between 4 and 5 days post-inoculation (Figure 1). CBoV-1 isolate was designated as MX-ABM30.

### 3.2. Amplification of CBoV-1 Genome

Using the designed primers, four PCR products of 1242 bp, 1408 bp, 1506 bp, and 1589 bp were obtained from isolate MX-ABM30 (Figure 2). The assembled sequence was 4973 bp in length, confirmed as CBoV-1, and covers all coding regions (CDS). The genes *NS1* (2325 nt, 775 aa), *NP1* (561 nt, 187 aa), *VP1* (2112 nt, 704 aa), and *VP2* (1716 nt, 572 aa) were identified. CBoV-1 sequence was deposited in GenBank under accession number PQ683622.

### 3.3. Sequence Analysis

A multiple alignment of complete genome sequences of CBoV, including isolate MX-ABM30, was performed. Based on the genetic distances, a dendrogram was constructed (Figure 3), revealing three distinct clades corresponding to subtypes CBoV-1, CBoV-2, and CBoV-3. Isolate MX-ABM30 clustered within the CBoV-1 clade, with 87.3–98.1% nucleotide identity, exhibiting a close genetic relationship with isolates from the United States, Portugal, Japan, and Italy, whereas isolate MX-ABM30 showed a nucleotide identity of 62% and 59% with the CBoV-2 and CBoV-3 clades, respectively.

A comparative analysis of the MX-ABM30 isolate against 17 CBoV-1 reference sequences revealed a total of 22 distinct nucleotide mutations within the *NS1* gene, four of which were missense mutations resulting in the amino acid substitutions K176R, K182N, P669S, and I680L. Six nucleotide mutations were identified in the *NP1* gene; however, all were silent mutations and therefore did not alter the amino acid sequence of the encoded protein. Additionally, a total of 38 nucleotide mutations were detected within the shared coding region of *VP1* and *VP2* genes. Among these, only two missense mutations were identified, resulting in amino acid substitutions S200G and S536A. In Table 3, we summarize the amino acid substitutions observed in isolate MX-ABM30 compared against 17 CBoV-1 sequences.

## 4. Discussion

Canine bocavirus type 1 (CBoV-1) is considered an emerging pathogen of interest due to its association with enteric and respiratory diseases in dogs, although the virus has also been associated with reproductive disorders, including neonatal mortality [13,14,15]. Despite its wide geographic distribution [14,28], CBoV-1 has not previously been reported in Mexico. In this study, CBoV-1 was successfully isolated from a clinical sample obtained from a dog with acute hemorrhagic diarrhea, and its genome was subsequently amplified and sequenced, providing the first genetic characterization of an isolated CBoV-1 strain in Mexico.

The successful isolation of CBoV-1 from a dog with acute hemorrhagic gastroenteritis provides evidence of active viral replication during clinical disease. However, the concurrent detection of CPV-2 and CCV prevents attribution of the clinical outcome to CBoV-1 alone. The fact that CBoV-1 was the sole virus isolated from the three present in the sample could be attributed to several factors such as low viral loads or reduced susceptibility of the cell line. Additionally, some viruses demand highly specific conditions for in vitro replication, and the absence of these can lead to latent or abortive infections that complicate isolation [36]. For instance, while CPV-2 isolation is standard practice, CCV presents a unique challenge, as the virus has not been successfully propagated in any established cell line to date [37]. Nevertheless, the isolation of CBoV-1 raises the possibility that it may contribute to disease as a negative co-factor. Viral coinfections have been associated with increased disease severity and mortality [38,39,40]. In CPV-2 and CCV coinfections, CCV has been shown to induce immunosuppression in the host by inhibiting the activation of the type I interferon (IFN-I) promoter, thereby facilitating CPV-2 replication and potentially contributing to increased clinical disease severity [41]. To date, immunosuppressive effects have not been demonstrated for CBoV; however, porcine bocavirus (PBoV) NP1 protein has been shown to suppress type I interferon (IFN-I) production [10]. These findings raise the possibility that CBoV could have similar effects on the host’s immune response that could influence disease severity during coinfections, although this hypothesis remains to be investigated.

The established CBoV-1 sequence from isolate MX-ABM30 (GenBank accession number PQ683622) showed 87.3–98.1% nucleotide identity with previously reported worldwide CBoV-1 sequences, suggesting relatively low genetic variability among the analyzed strains, as previously described [14]. However, this finding may be attributed to the limited availability of complete CBoV-1 genome sequences, which may lead to an underestimation of the true genetic diversity of the virus.

Phylogenetic analysis revealed a close genetic relationship between isolate MX-ABM30 and sequences reported from Brazil, Canada, the United States of America, Portugal, Japan, Korea, and Italy, supporting the widespread distribution of CBoV-1 described in previous studies [14,28]. The clustering of MX-ABM30 within a separate subclade, although with limited bootstrap support, may indicate the presence of a genetically differentiated lineage circulating in Mexico; however, further molecular epidemiological studies are needed to better understand the genetic diversity and evolutionary patterns of CBoV-1 in Mexico.

Comparative analysis of isolate MX-ABM30 identified four amino acid substitutions in the *NS1* gene: K176R, K182N, P669S, and I680L. Notably, substitutions K176R and K182N were not observed in the other sequences analyzed, suggesting that these mutations may be characteristic of MX-ABM30. Substitutions P669S and I680L were also found in sequences from China, Japan, and Portugal, whereas variability at residue 680 was observed among sequences from several countries, suggesting that this position may be genetically variable. Although the *NS1* gene plays a key role in viral replication, there is currently no evidence linking specific mutations to changes in the pathogenesis of CBoV-1 [13,28]. In contrast, no amino acid substitutions were identified in the *NP1* gene. The genetic conservation of NP1 may be attributed to its critical role in regulating capsid protein expression through pre-mRNA processing [6].

Within the shared coding region of *VP1* and *VP2* genes, isolate MX-ABM30 exhibited two amino acid substitutions: S200G and S536A, which were also identified in sequences reported from Brazil, Canada, China, and Italy. Interestingly, substitution S200G is located within the VR-III region at the twofold symmetry axis of the capsid. Although this axis is described as a depression on the capsid surface in most bocaviruses, in CBoV-1 it forms a protrusion due to the presence of additional amino acids in VR-III compared with other bocaviruses [3,5]. Similarly, substitution S536A is located at the twofold symmetry axis within the variable region VR-IX, which exhibits an increased radial surface in CBoV-1 compared with Human bocavirus (HBoV) and Bovine parvovirus (BPV) [5]. Because these substitutions are in surface-exposed regions of the capsid, they may be involved in virus–host interactions and potentially contribute to viral adaptation to different host species or changes in the antigenic properties of CBoV-1. However, further studies are needed to determine their functional impact on CBoV-1 pathogenesis and antigenicity.

Overall, this study provides the first evidence of CBoV-1 isolation and genomic characterization from a clinical case in Mexico. Although a single clinical case cannot define the broader epidemiology or clinical impact of the virus, the genomic data obtained here contribute to the current knowledge of CBoV-1 genetic diversity. Because known enteric pathogens (CPV-2 and CCV) were also present, the specific clinical impact of CBoV-1 in this patient cannot be definitively determined. Ultimately, this study provides a foundation for future epidemiological surveys and in vivo pathogenic studies.

## 5. Conclusions

This study reports the isolation and genome characterization of Bocaparvovirus carnivoran 1 (CBoV-1) in Mexico, obtained from a dog with acute hemorrhagic diarrhea. The isolate MX-ABM30 showed high genetic similarity to CBoV-1 strains circulating in other countries and exhibited unique amino acid substitutions in the NS1 protein. Although broad epidemiological conclusions cannot be drawn from one case, the genomic data presented here provides valuable initial insights into the genetic diversity of CBoV-1. Given the frequent presence of viral co-infections, these findings underscore the importance of investigating the complex canine enteric virome to clarify the specific contribution of CBoV-1 in acute hemorrhagic gastroenteritis.

## Figures and Tables

**Figure 1:**
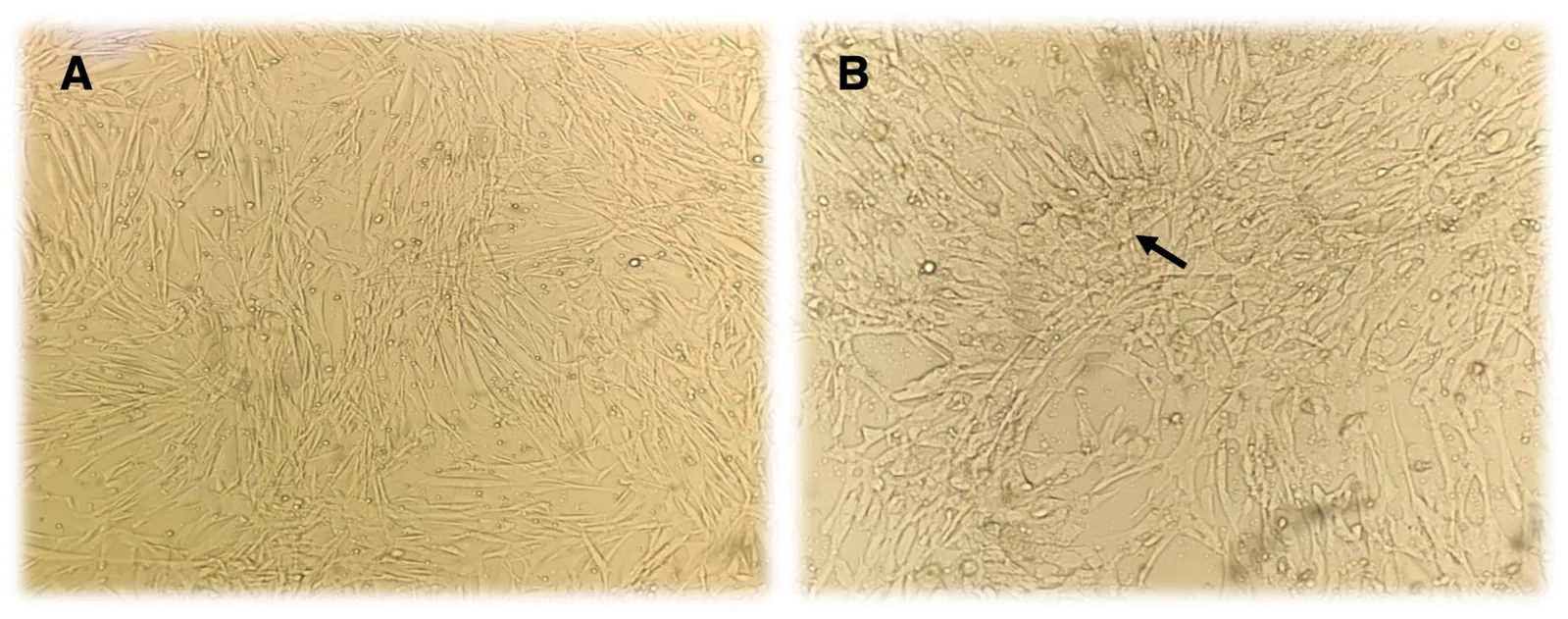
Cytopathic effect of CBoV-1 in MDCK cells. (**A**) Mock MDCK cells (control). (**B**) MDCK cells infected with CBoV-1 at 5 days post-infection. The arrow shows characteristic rounding and detachment. Magnification: 40×.

**Figure 2:**
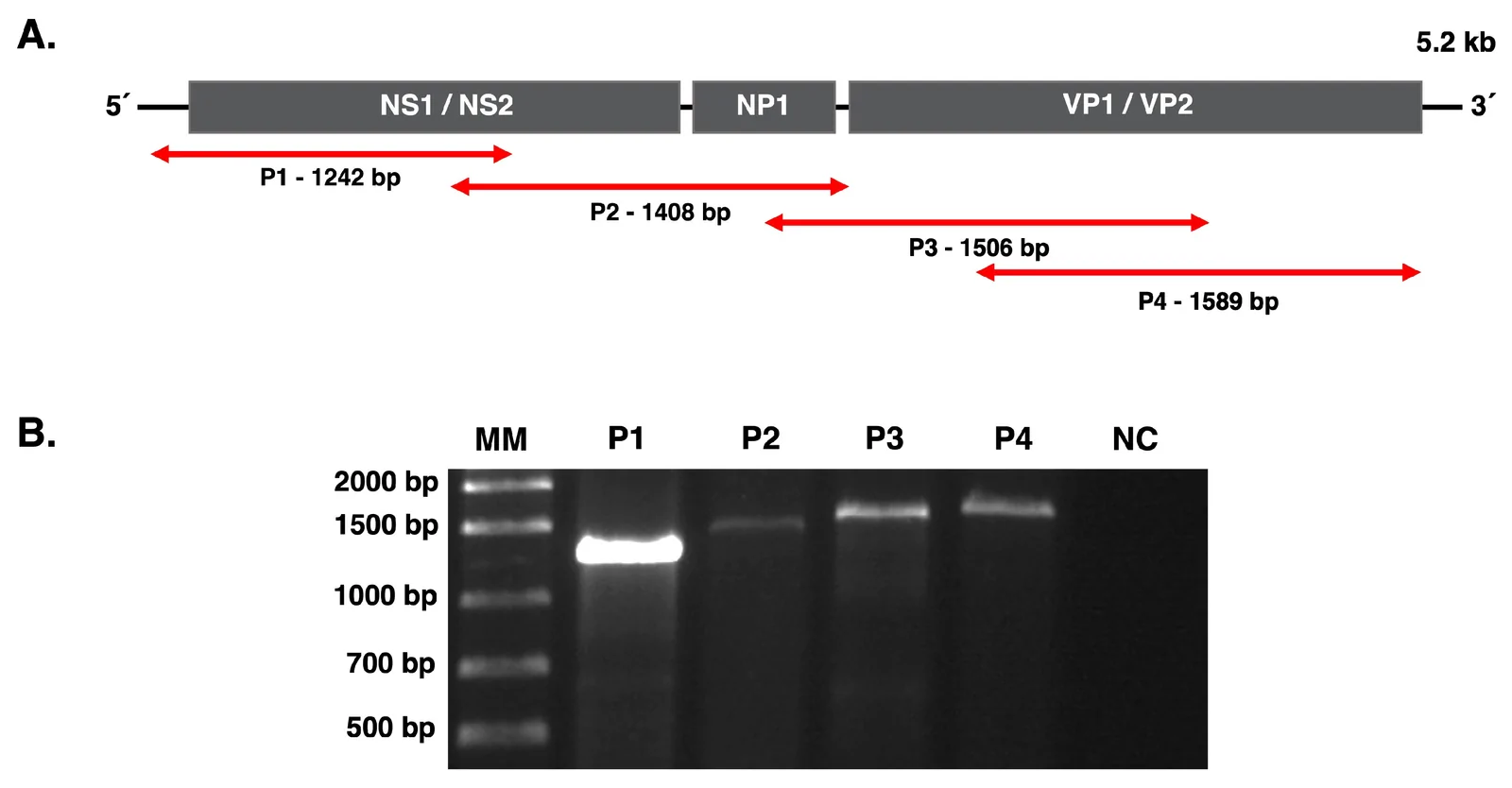
Whole-genome amplification of CBoV-1. (**A**) Schematic representation of the CBoV-1 genome and the four overlapping PCR fragments (P1–P4) used for whole-genome amplification. (**B**) Electrophoresis gel of the four overlapping amplicons spanning the entire CBoV-1 genome from isolate MX-ABM30, as shown in panel A. Expected amplicon sizes were approximately 1242 bp (P1), 1408 bp (P2), 1506 bp (P3), and 1589 bp (P4). MM, molecular weight marker. NC, negative control (DEPC water).

**Figure 3:**
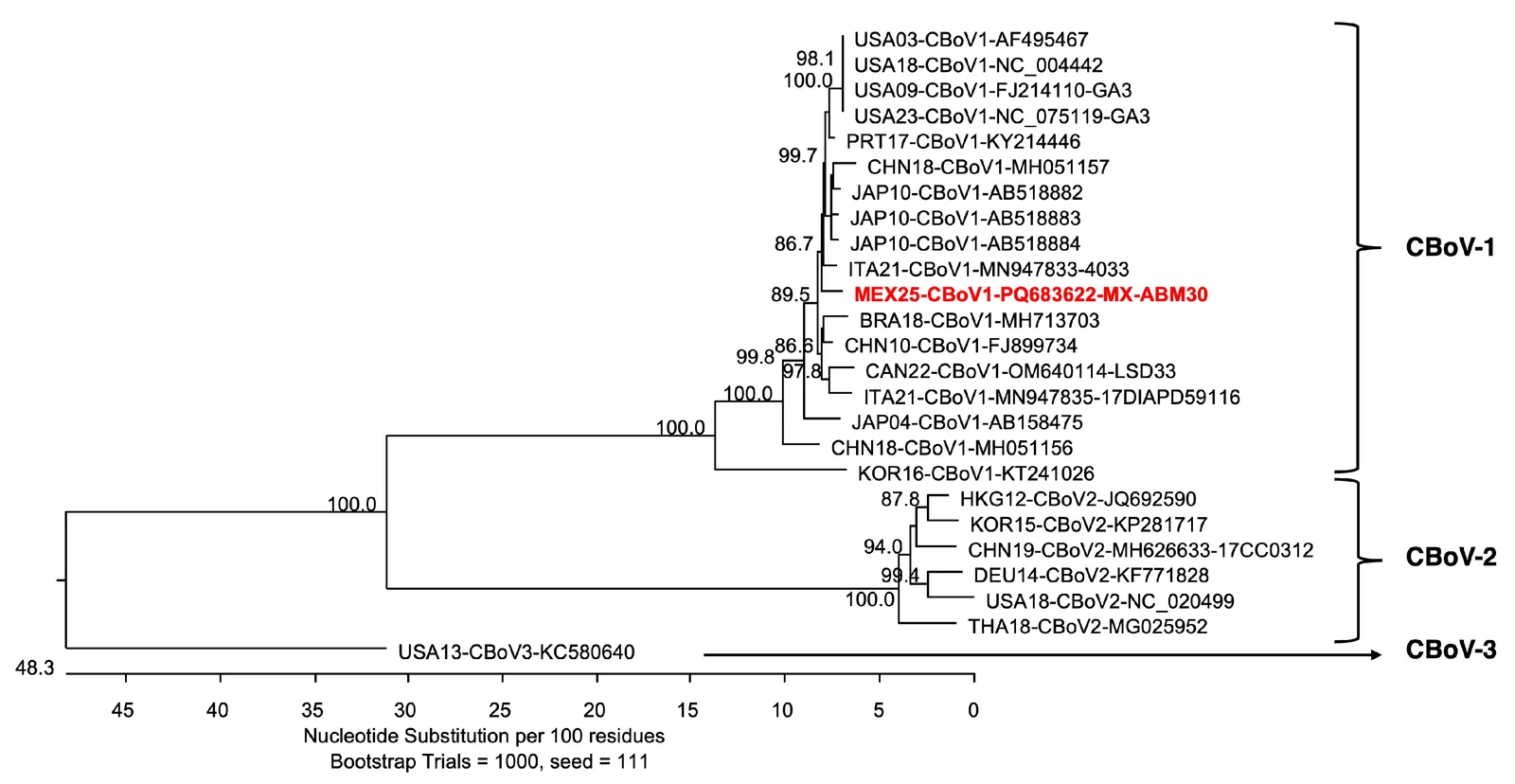
Dendrogram based on complete genome sequences of CBoV. Isolate MX-ABM30 is highlighted in red. Bootstrap values ≥ 70 are displayed at the branching nodes.

**Table 1:** Primers designed for clinical molecular detection of CPV-2, CCV, CBoV-1, and CBoV-2.

Primer ID	Target Gene	Sequence (5′-3′)	Nt. Position	GenBank Reference	Product Size (bp)
CPV2748F	CPV-2 (VP2)	CCGGTGCAGGACAAGTAAAAAG	2748	NC_001539	1779
CPV4527R		CTAGGTGCTAGTTGAGATTTTTCAT	4527		
CCV/CAP/1517	CCV (Cap)	ATCTTGTARCATTYCCTCTTCC	1517	JQ821392	393
CCV/CAP/1908		CCGGGCAAGTCGSCG	1908		
CBoV/NP1/2305	CBoV-1 (NP1)	GGAGCCGGAGTCGSAG	2305	NC_004442	273
CBoV1/NP1/2678		TCATTAAAAATCCAGTCAGTCCCA	2678		
CBoV/NP1/2305	CBoV-2 (NP1)	GGAGCCGGAGTCGSAG	2305	KY038922	215
CBoV2/NP1/2520		GTGCCAGTAAAAGCCACACA	2520		

**Table 2:** Primers designed to amplify the complete genome of CBoV-1.

Primer ID	Sequence (5′-3′)	Nt. Position	GenBank Reference	Product Size (bp)
CBoV1/1-25/FWD	TGGAGGACGGAAGTGTGATTC	25	NC_004442	1242
CBoV1/1-1266/REV	CGGCGCACCCATTTACAAG	1266		
CBoV1/2-1117/FWD	TGCCTAAGGTACCTGAATCTTCTTG	1117	NC_004442	1408
CBoV1/2-2524/REV	TGGAGGACGATTGGTTCCTG	2524		
CBoV1/3-2157/FWD	ACAGGTGAGATCCTTAATCACAATG	2157	NC_004442	1506
CBoV1/3-3662/REV	TGAGACGTTGCCAGTCTTGT	3662		
CBoV1/4-3507/FWD	CACGACAGTGGTATGCACCT	3507	NC_004442	1589
CBoV1/4-5095/REV	AAACACACCCAGTGGTGACG	5095		

**Table 3:** Amino acid substitutions in CBoV-1 sequences.

GenBank Reference	Country	Amino Acid Position (NS1)				Amino Acid Position (VP2)	
		176	182	669	680	200	536
MH711303	Brazil	K	K	P	S	G	A
OM640114	Canada	K	K	P	S	G	A
FJ899734	China	K	K	S	I	S	A
MH051156	China	K	K	P	N	G	A
MH051157	China	K	K	S	I	S	S
MN947833	Italy	K	K	P	I	S	S
MN947835	Italy	K	K	P	S	G	A
AB158475	Japan	K	K	S	L	G	S
AB518882	Japan	K	K	S	I	S	S
AB518883	Japan	K	K	S	I	S	S
AB518884	Japan	K	K	S	I	S	S
**PQ683622**	**Mexico**	**R**	**N**	**S**	**L**	**G**	**A**
KY214446	Portugal	K	K	S	I	S	S
AF495467	USA	K	K	P	S	S	S
FJ214110	USA	K	K	P	S	S	S
NC_004442	USA	K	K	P	S	S	S
NC_075119	USA	K	K	P	S	S	S

The row in bold corresponds to CBoV-1 isolate MX-ABM30 (PQ683622). Novel amino acid substitutions are highlighted in red.

## Data Availability

All relevant data are provided in the present manuscript. Obtained sequence have been submitted to GenBank (https://www.ncbi.nlm.nih.gov/nucleotide/), accessed on 9 February 2025.

## Supplementary Materials

The following supporting information can be downloaded at: https://www.mdpi.com/article/10.3390/v18101064/s1. Table S1: CBoV whole-genome sequence data used in this study.

## Abbreviations

The following abbreviations are used in this manuscript:

aa	Amino acid
bp	Base pair
°C	Celsius
CBoV	*Canine bocavirus*
CCV	*Canine circovirus*
CCoV	*Canine coronavirus*
CO_2_	Carbon dioxide
CPE	Cytopathic effect
CPV-2	*Canine parvovirus type 2*
DEPC	Diethyl pyrocarbonate
DMEM	Dulbecco’s Modified Eagle Medium
DNA	Deoxyribonucleic acid
FBS	Fetal bovine serum
FWD	Forward
g	g-force unit
G	Glycine
I	Isoleucine
K	Lysine
L	Leucine
µm	Micron
µl	Microliter
MDCK	Madin–Darby canine kidney
min	Minutes
N	Asparagine
NS	Non-structural protein
Nt	Nucleotide
ORF	Open Reading Frame
P	Proline
PCR	Polymerase chain reaction
R	Arginine
REV	Reverse
s	Seconds
S	Serine
VP	Structural protein
