# Supplementary material for: First Isolation and Genome Characterization of Bocaparvovirus carnivoran 1 (CBoV-1) from a Dog with Acute Hemorrhagic Diarrhea in Mexico
Source: Viruses. 2026 Sep 25;18(10):1064. doi: 10.3390/v18101064 (PMC13709931; doi:10.3390/v18101064)
Supplement: Supplementary file 1 [file viruses-18-01064-s001.zip › viruses-4565099-supplementary.pdf]

Table S1: CBoV whole genome sequences data used in this study.

GenBank Reference	Isolate or strain	Genotype	Host	Country
MH713703	Paraiba	CBoV-1	Domestic dog	Brazil
OM640114	LSD33	CBoV-1	Domestic dog	Canada
FJ899734	SH1	CBoV-1	Domestic dog	China
MH051156	CDK47/2017	CBoV-1	Domestic dog	China
MH051157	CQY9/2016	CBoV-1	Domestic dog	China
MN947833	4033/Italy/2012	CBoV-1	Domestic dog	Italy
MN947835	59116/Italy/2017	CBoV-1	Domestic dog	Italy
AB158475	HM-6	CBoV-1	Domestic dog	Japan
AB518882	08-017	CBoV-1	Domestic dog	Japan
AB518883	97-047	CBoV-1	Domestic dog	Japan
AB518884	97-114	CBoV-1	Domestic dog	Japan
KY214446	South Douro	CBoV-1	Domestic dog	Portugal
AF495467	NA	CBoV-1	Domestic dog	USA
FJ214110	GA 3	CBoV-1	Domestic dog	USA
NC_004442	NA	CBoV-1	Domestic dog	USA
NC_075119	GA 3	CBoV-1	Domestic dog	USA
MH626633	17CC0312	CBoV-2	Domestic dog	China
KF771828	F13000791S	CBoV-2	Domestic dog	Germany
JQ692590	HK882U	CBoV-2	Domestic dog	Hong Kong
KP281717	14D142	CBoV-2	Domestic dog	South Korea
MG025952	TH-2016	CBoV-2	Domestic dog	Thailand
NC_020499	Con-161	CBoV-2	Domestic dog	USA
KC580640	UCD	CBoV-3	Domestic dog	USA
